# Neuroticism personality traits are linked to adverse cardiovascular phenotypes in the UK Biobank

**DOI:** 10.1093/ehjci/jead166

**Published:** 2023-07-13

**Authors:** Adil Mahmood, Judit Simon, Jackie Cooper, Theodore Murphy, Celeste McCracken, Juan Quiroz, Liliana Laranjo, Nay Aung, Aaron Mark Lee, Mohammed Y Khanji, Stefan Neubauer, Zahra Raisi-Estabragh, Pal Maurovich-Horvat, Steffen E Petersen

**Affiliations:** William Harvey Research Institute, NIHR Barts Biomedical Research Centre, Queen Mary University of London, Charterhouse Square, London EC1M 6BQ, UK; MTA-SE Cardiovascular Imaging Research Group, Department of Radiology, Medical Imaging Centre, Semmelweis University, Budapest, Hungary; William Harvey Research Institute, NIHR Barts Biomedical Research Centre, Queen Mary University of London, Charterhouse Square, London EC1M 6BQ, UK; Department of Cardiology and Cardiovascular Imaging, Beacon Hospital, Dublin, Ireland; William Harvey Research Institute, NIHR Barts Biomedical Research Centre, Queen Mary University of London, Charterhouse Square, London EC1M 6BQ, UK; Division of Cardiovascular Medicine, Radcliffe Department of Medicine, University of Oxford, National Institute for Health Research Oxford Biomedical Research Centre, Oxford University Hospitals NHS Foundation Trust, Oxford OX3 9DU, UK; Centre for Big Data Research in Health (CBDRH), The University of New South Wales (UNSW), Sydney, Australia; Faculty of Medicine and Health, Westmead Applied Research Centre (WARC), University of Sydney, Australia; William Harvey Research Institute, NIHR Barts Biomedical Research Centre, Queen Mary University of London, Charterhouse Square, London EC1M 6BQ, UK; Barts Heart Centre, St Bartholomew’s Hospital, Barts Health NHS Trust, West Smithfield, EC1A 7BE, London, UK; William Harvey Research Institute, NIHR Barts Biomedical Research Centre, Queen Mary University of London, Charterhouse Square, London EC1M 6BQ, UK; William Harvey Research Institute, NIHR Barts Biomedical Research Centre, Queen Mary University of London, Charterhouse Square, London EC1M 6BQ, UK; Barts Heart Centre, St Bartholomew’s Hospital, Barts Health NHS Trust, West Smithfield, EC1A 7BE, London, UK; Division of Cardiovascular Medicine, Radcliffe Department of Medicine, University of Oxford, National Institute for Health Research Oxford Biomedical Research Centre, Oxford University Hospitals NHS Foundation Trust, Oxford OX3 9DU, UK; William Harvey Research Institute, NIHR Barts Biomedical Research Centre, Queen Mary University of London, Charterhouse Square, London EC1M 6BQ, UK; Barts Heart Centre, St Bartholomew’s Hospital, Barts Health NHS Trust, West Smithfield, EC1A 7BE, London, UK; MTA-SE Cardiovascular Imaging Research Group, Department of Radiology, Medical Imaging Centre, Semmelweis University, Budapest, Hungary; William Harvey Research Institute, NIHR Barts Biomedical Research Centre, Queen Mary University of London, Charterhouse Square, London EC1M 6BQ, UK; Barts Heart Centre, St Bartholomew’s Hospital, Barts Health NHS Trust, West Smithfield, EC1A 7BE, London, UK; Health Data Research UK, London, UK; Alan Turing Institute, London, UK

**Keywords:** neuroticism, mental health, cardiovascular magnetic resonance, cardiac morphology, cardiac function, cardiovascular remodelling

## Abstract

**Aims:**

To evaluate the relationship between neuroticism personality traits and cardiovascular magnetic resonance (CMR) measures of cardiac morphology and function, considering potential differential associations in men and women.

**Methods and results:**

The analysis includes 36 309 UK Biobank participants (average age = 63.9 ± 7.7 years; 47.8% men) with CMR available and neuroticism score assessed by the 12-item Eysenck Personality Questionnaire-Revised Short Form. CMR scans were performed on 1.5 Tesla scanners (MAGNETOM Aera, Siemens Healthcare, Erlangen, Germany) according to pre-defined protocols and analysed using automated pipelines. We considered measures of left ventricular (LV) and right ventricular (RV) structure and function, and indicators of arterial compliance. Multivariable linear regression was used to estimate association of neuroticism score with individual CMR metrics, with adjustment for age, sex, obesity, deprivation, smoking, diabetes, hypertension, hypercholesterolaemia, alcohol use, exercise, and education. Higher neuroticism scores were associated with smaller LV and RV end-diastolic volumes, lower LV mass, greater concentricity (higher LV mass to volume ratio), and higher native T1. Greater neuroticism was also linked to poorer LV and RV function (lower stroke volumes) and greater arterial stiffness. In sex-stratified analyses, the relationships between neuroticism and LV stroke volume, concentricity, and arterial stiffness were attenuated in women. In men, association (with exception of native T1) remained robust.

**Conclusion:**

Greater tendency towards neuroticism personality traits is linked to smaller, poorer functioning ventricles with lower LV mass, higher myocardial fibrosis, and higher arterial stiffness. These relationships are independent of traditional vascular risk factors and are more robust in men than women.

## Graphical Abstract

**Graphical Abstract:**
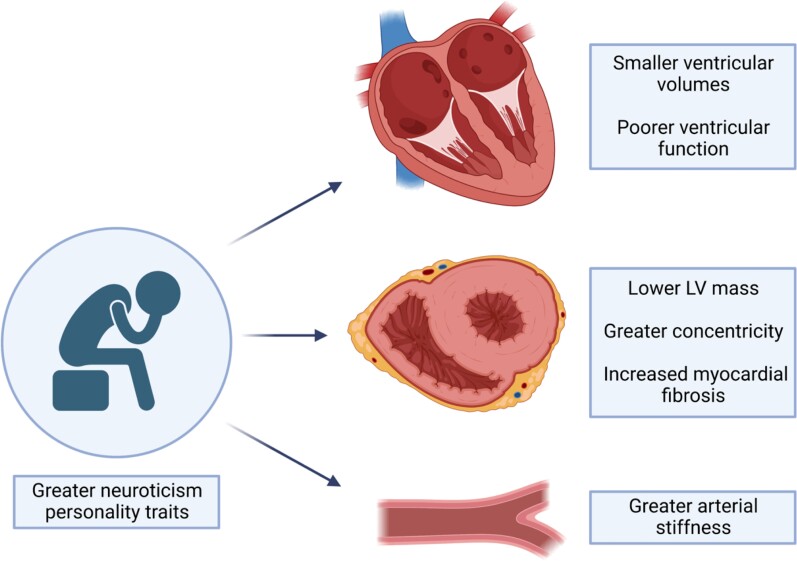

**See the editorial comment for this article ‘Personality traits and cardiovascular diseases: is it about ‘don't worry, be happy', or is this a deeper underlying problem?’, by O. A. Smiseth and T. C. Gillebert, https://doi.org/10.1093/ehjci/jead185.**

## Introduction

The burden of mental health conditions is increasing worldwide. In the last ten years, there has been a 13% increase in the global burden of mental illness and ∼20% of the world’s children and adolescents reported to have a mental health condition.^1^ The relationship between mental wellbeing and cardiovascular health is widely recognised, encompassing many contributing factors including greater socio-economic deprivation, adverse health behaviours and the metabolic effects of anti-psychotic medications.^2^ More recently, however, emerging evidence has suggested causal links between certain mental health conditions and cardiovascular risk independent of these potential confounding factors.^3–5^

Neuroticism is a fundamental personality trait encompassing characteristics such as unstable moods, excessive worrying, anxiety, irritability, self-consciousness, and sadness. The negative emotional states associated with neuroticism have been linked to increased risk for psychiatric illness such as depression and anxiety disorder^6,7^ and a range of cardiovascular disease (CVD) outcomes.^8–11^ Previous work aiming to elucidate the pathways driving these relationships has demonstrated association of high anxiety levels and suppressed anger with greater arterial stiffness.^12,13^ Vecsey-Nagy and colleagues demonstrated an affective temperament to be an independent predictor of severe coronary artery disease (CAD) when assessing patients referred for computed tomography coronary angiography.^14^ Furthermore, a recent genetic study has indicated a polygenic overlap between neuroticism and CAD and CVD risk factors, suggesting genetic factors may also contribute to the underlying pathology.^15^

At the same time, there are well-established sex-based differences in the prevalence of mental health conditions and CVD. For instance, while men have a higher propensity of developing antisocial personality disorder and substance abuse, women are more likely to be diagnosed with major depression.^16^ Similarly, men have a higher risk of CAD at younger ages compared to women,^17^ whereas women are more likely to develop stroke^18^ and heart failure.^19,20^

Cardiovascular imaging captures early pre-clinical alterations of cardiac structure and function. Evaluating the association of neuroticism personality traits with cardiovascular phenotypes can provide insight into the associated cardiovascular risk and the mechanisms through which these risks may be mediated. Such analyses have not been previously undertaken in large population-based cohorts. We examined associations between neuroticism scores and cardiovascular magnetic resonance (CMR) measures of cardiac structure and function in over 30 000 UK Biobank participants, considering potential differential relationships in men and women.

## Methods

### Study population

The UK Biobank is a prospective cohort study which collected questionnaire data, physical measurements, and biological samples from over half a million 40–69-year-old individuals in the United Kingdom recruited between 2006 and 2010.^21^ The UK Biobank Imaging Study, which is ongoing, aims to scan 100 000 of the original participants. The imaging protocol includes detailed CMR.^22^ Our analysis includes all UK Biobank participants with CMR data and neuroticism score available. We excluded individuals with pre-existing CVD (heart failure, angina, prior myocardial infarction, stroke).

### Ethics

This study was covered by the ethical approval for UK Biobank studies from the NHS National Research Ethics Service on 17 June 2011 (Ref 11/NW/0382) and extended on 10 May 2016 (Ref 16/NW/0274) and on 18 June 2021 (Ref 21/NW/0157) with written informed consent obtained from all participants.

### CMR protocol and image analysis

The UK Biobank CMR protocol is detailed elsewhere.^23,24^ Briefly, all examinations were performed at imaging visit on a 1.5 Tesla scanner (MAGNETOM Aera, Syngo Platform VD13A, Siemens Healthcare, Erlangen, Germany). For cardiac function, long-axis cines and a complete short-axis stack of balanced steady-state free precession cines were acquired covering the left and right ventricle. Image analysis was performed using fully automated pipelines with in-built quality control, previously developed and validated in the UK Biobank.^25,26^

We included the following CMR metrics in our analysis: left and right ventricular end-diastolic volumes (LVEDV, RVEDV), left and right ventricular stroke volumes (LVSV, RVSV), left ventricular mass (LVM), left ventricular mass-to-volume ratio (LVM/LVEDV), left ventricular global longitudinal strain, left ventricular global function index, and native T1.

We also considered two measures of arterial stiffness: aortic distensibility (AoD), a CMR measure derived using this pipeline,^27^ and arterial stiffness index (ASI), a measure of large artery stiffness calculated using the pulse waveform obtained at the finger with an infra-red sensor (PulseTrace PCA 2TM, CareFusion, USA).

### Measurement of neuroticism

Neuroticism was assessed at baseline recruitment using the 12 items of the neuroticism scale from the Eysenck Personality Questionnaire-Revised Short Form (EPQ-R-S), as part of the UK Biobank assessment.^28^ The score questions are summarised in Supplementary data online, *Table S1*, with each answer of ‘yes’ adding 1 point to the total score. Previous analysis of neuroticism scores between the EPQ-R-S and NEO-Fiver Factor Inventory, the most widely used scale internationally, demonstrated an 85% correlation thus conferring high concurrent validity.^29,30^ In analysis, we assessed differences in CMR associated with one standard deviation (1SD) increase in neuroticism score, and differences observed in participants with moderate neuroticism (scores 3–5) and high neuroticism (scores 6+) compared to those with low neuroticism (scores 0–1).

### Ascertainment of covariates

We sought to determine the association of neuroticism with CMR phenotypes independent of traditional vascular risk factors, and selected model covariates on this basis. Ethnicity and sex were self-reported at baseline. Townsend deprivation index, a socio-economic measure of deprivation, was calculated prior to participants joining the UK Biobank based on area of residence. Age was as recorded at imaging. Alcohol intake frequency (never, special occasions only, 1–3 times per month, 1–2 times per week, 3–4 times per week, and daily or almost daily), smoking status (never smoker and previous or current smoker), and educational level were self-reported. Body mass index (BMI) was calculated from height and weight measures taken at imaging. Diabetes, hypertension, and hypercholesterolaemia status, at imaging, were defined from a combination of self-report and record of the diagnosis in any of the linked databases. Total cholesterol level was as per blood biochemistry taken at baseline. Physical activity was calculated by weighting different types of activity (walking, moderate, vigorous) by its energy requirements as reported at imaging.^31^ The UK Biobank protocol is publicly available.^32^

### Statistical analysis

Statistical analysis was performed using R 4.1.0. Summary statistics were presented as means with SD or medians with interquartile range for continuous variables and percentages with frequency for categorical data. Neuroticism score was the exposure variable and each CMR metric was inserted in turn as the outcome variable. We estimated the association between neuroticism and CMR metrics using multivariable linear regression models, adjusting for the following covariates: age, sex, BMI, Townsend deprivation score, smoking, diabetes, hypertension, hypercholesterolaemia, total cholesterol level, alcohol use, exercise, and education. Models were fitted for the whole cohort and then stratified by sex. Residual plots were used to check for heteroscedasticity and extreme observations. We report associations with neuroticism as standardised betas to allow cross-comparability of effect sizes with CMR metrics, which lie on different measurement scales, with corresponding 95% confidence intervals (CI) and *P*-values. Significance thresholds were adjusted for multiple testing using a false discovery rate of 5%.^33^

## Results

### Baseline characteristics

A total of 37 817 individuals had CMR imaging data and completed the neuroticism scale. Of these, 1508 individuals were excluded on account of pre-existing CVD. The remaining 36 309 participants were included in the primary analysis. The mean age was 63.9 years with a greater proportion of females (52.2%). Hypertension, diabetes mellitus, and hypercholesterolaemia were present in 26.7%, 5.3%, and 23.1% of participants, respectively, and the mean BMI was 26.4 kg/m^2^ (*Table 1*). The CMR parameters for the study population are summarised in *Table 2*. Baseline characteristics, stratified by neuroticism score, are presented in *Table 3*. The majority of participants (41.9%) were in the lowest tertile of the neuroticism score. In comparison to those in the highest tertile, those in the lower tertile of the neuroticism score were older, more likely to be male, less likely to smoke, and were more affluent. The mean and median neuroticism scores were 3.8 (±3.2) and 3 [1–6], respectively, and the distribution of scores are presented in *Supplementary data online, Figure S1*.

**Table 1:** Baseline characteristics by sex

Variable	Whole cohort	Male	Female
	*n* = 36 309	*n* = 17 340 (47.8%)	*n* = 18 969 (52.2%)
Age (years)	63.9 ± 7.7	64.5 ± 7.8	63.3 ± 7.5
Smoking (current), *n* (%)	1203 (3.3)	687 (4.0)	516 (2.7)
Diabetes, *n* (%)	1933 (5.3)	1213 (7.0)	720 (3.8)
Hypertension, *n* (%)	9711 (26.7)	5621 (32.4)	4090 (21.6)
Hypercholesterolaemia, *n* (%)	8397 (23.1)	5308 (30.6)	3089 (16.3)
Total cholesterol level (mmol/L)	5.74 ± 1.1	5.62 ± 1.1	5.86 ± 1.1
Alcohol>= 3 times/week, *n* (%)	16 488 (45.7)	9151 (53.2)	7337 (39.0)
BMI (kg/m^2^)	26.4 ± 4.4	26.9 ± 3.9	26.0 ± 4.7
Townsend deprivation score	−2.62 [−3.88, −0.51]	−2.69 [−3.93, −0.59]	−2.56 [−3.84, −0.45]
IPAQ score (METs/week)	2190 [1124, 3930]	2154 [1124,3902]	2226 [1122,3971]
Degree or professional qualification, *n* (%)	24 258 (67.6)	11 926 (68.8)	12 602 (66.5)

Mean ± standard deviation or median [interquartile range] for continuous variables. Case numbers (percentages) for categorical variables. BMI, body mass index; IPAQ, international physical activity questionnaire.

**Table 2:** Baseline CMR measures by sex

CMR Parameter	Whole cohort	Male	Female
	*n* = 36 309	*n* = 17 340	*n* = 18 969
LVEDV (mL)	147.1 ± 33.3	167.2 ± 31.5	128.8 ± 22.6
LVSV (mL)	87.0 ± 19.1	96.4 ± 19.3	78.5 ± 14.4
LVM (g)	85.9 ± 22.2	102.4 ± 18.5	70.8 ± 12.4
LVM/LVEDV (g/mL)	0.59 ± 0.09	0.62 ± 0.09	0.56 ± 0.08
LVGLS (%)	−18.5 ± 2.7	−17.8 ± 2.6	−19.1 ± 2.63
LVGFI (%)	0.48 ± 0.07	0.45 ± 0.06	0.50 ± 0.06
Native T1 (ms)	932.0 ± 35.3	919.2 ± 32.5	943.7 ± 33.7
AoD (10^−3^/mmHg)	2.51 ± 1.59	2.50 ± 1.45	2.53 ± 1.7
ASI (m/s)	9.6 ± 3.0	10.1 ± 2.9	9.2 ± 3.0
RVEDV (mL)	156.3 ± 37.1	180.6 ± 33.4	134.0 ± 24.1
RVSV (mL)	88.6 ± 20.2	99.1 ± 19.9	79.0 ± 14.9

Mean ± standard deviation. AoD, aortic distensibility; ASI, arterial stiffness index; CMR, cardiovascular magnetic resonance; LVEDV, left ventricular end-diastolic volume; LVGFI, left ventricular global function index; LVGLS, left ventricular global longitudinal strain; LVM, left ventricular mass; LVSV, left ventricular stroke volume; RVEDV, right ventricular end-diastolic volume; RVSV, right ventricular stroke volume.

**Table 3:** Baseline characteristics by neuroticism score

Variable	Tertile of neuroticism score		
	0–2	3–5	6+
*n*	15 209 (41.9%)	10 698 (29.5%)	10 402 (28.6%)
Age (years)	64.8 ± 7.7	63.8 ± 7.7	62.7 ± 7.5
Male, *n* (%)	8561 (56.3)	4675 (43.7)	4104 (39.5)
Smoking (current), *n* (%)	447 (3.0)	337 (3.2)	419 (4.1)
Diabetes, *n* (%)	802 (5.3)	573 (5.4)	558 (5.4)
Hypertension, *n* (%)	3987 (26.2)	2862 (26.8)	2862 (27.5)
Hypercholesterolaemia, *n* (%)	3545 (23.3)	2446 (22.9)	2406 (23.1)
Total cholesterol (mmol/L)	5.75 ± 1.1	5.75 ± 1.1	5.73 ± 1.1
Alcohol ≥ 3 times/week, *n* (%)	7122 (47.2)	4923 (46.3)	4443 (43.1)
BMI (kg/m^2^)	26.4 ± 4.2	26.3 ± 4.4	26.6 ± 4.6
Townsend deprivation score	−2.70 [−3.93, −0.67]	−2.63 [−3.89, −0.54]	−2.49 [−3.82, −0.24]
IPAQ score (METs/week)	2293 [1182, 4044]	2187 [1158,3906]	2038 [1004,3750]
Degree or professional qualification, *n* (%)	10 696 (70.4)	7227 (67.6)	6605 (63.5)

Mean ± standard deviation or median [interquartile range] for continuous variables. Case numbers (percentages) for categorical variables. BMI, body mass index; IPAQ, international physical activity questionnaire.

### Associations between neuroticism and CMR metrics for whole cohort

For the whole cohort, in fully adjusted models, each 1SD increase in neuroticism score was associated with smaller LVEDV [standardised beta = −0.024, 95% CI = (−0.033 to −0.015)] and RVEDV (−0.027 [−0.036 to −0.019]), lower LVSV [−0.018 (−0.027 to −0.008)] and RVSV [−0.021 (−0.030 to −0.011)], smaller LVM [−0.014 (−0.021 to −0.007)], and greater concentricity [LVM/LVEDV, 0.011 (0.002 to 0.021)]. Furthermore, higher neuroticism score was associated with higher native T1 [0.019 (0.008 to 0.029)] and greater ASI [0.019 (0.007 to 0.032)]. We observed evidence of statistically significant sex interaction for associations with LVM/LVEDV and native T1 values. (*Table 4*).

**Table 4:** Associations of cardiovascular phenotypes with increasing neuroticism score

Metric	Whole cohort	Men	Women	Neuroticism × sex interaction
LVEDV (mL)	−0.024^a^	−0.033^a^	−0.024^a^	−0.017
	[−0.033, −0.015]	[−0.048, −0.018]	[−0.039, −0.009]	[−0.034, 0.000]
	5.84 × 10^−8^	1.65 × 10^−5^	1.21 × 10^−3^	0.055
LVSV (mL)	−0.018^a^	−0.024^a^	−0.014	−0.015
	[−0.027, −0.008]	[−0.039, −0.009]	[−0.029, 0.000]	[−0.033, 0.004]
	4.14 × 10^−4^	2.81 × 10^−3^	0.06	0.13
LVM (g)	−0.014^a^	−0.019^a^	−0.018^a^	0.004
	[−0.021, −0.007]	[−0.033, −0.005]	[−0.032, −0.004]	[−0.010, 0.018]
	7.22 × 10^−5^	4.48 × 10^−3^	9.46 × 10^−3^	0.570
LVM/LVEDV (g/mL)	0.011^a^	0.018^a^	0.006	0.028^a^
	[0.002, 0.021]	[0.003, 0.033]	[−0.008, 0.021]	[0.009, 0.048]
	0.02	0.02	0.39	0.004
LVGLS (%)	0.014	0.010	0.019	−0.005
	[−0.001, 0.028]	[−0.012, 0.031]	[−0.001, 0.040]	[−0.034, 0.023]
	0.07	0.37	0.07	0.71
LVGFI (%)	0.002	−0.004	0.007	−0.013
	[−0.010, 0.014]	[−0.023, 0.014]	[−0.011, 0.025]	[−0.037, 0.011]
	0.78	0.64	0.46	0.29
Native T1 (ms)	0.019^a^	0.009	0.030^a^	−0.037^a^
	[0.008, 0.029]	[−0.007, 0.025]	[0.014, 0.046]	[−0.058, −0.015]
	7.25 × 10^−4^	0.28	1.80 × 10^−4^	0.0008
AoD (10–3/mmHg)	−0.004	−0.006	−0.003	−0.014
	[−0.019, 0.010]	[−0.026, 0.015]	[−0.023, 0.018]	[−0.043, 0.015]
	0.57	0.58	0.78	0.35
ASI (m/s)	0.019^a^	0.030^a^	0.010	0.020
	[0.007, 0.032]	[0.012, 0.047]	[−0.007, 0.027]	[−0.004, 0.044]
	1.71 × 10^−3^	7.33 × 10^−4^	0.25	0.10
RVEDV (mL)	−0.027^a^	−0.034^a^	−0.037^a^	−0.007
	[−0.036, −0.019]	[−0.049, −0.019]	[−0.051, −0.022]	−0.023, 0.010]
	1.22 × 10^−10^	1.11 × 10^−5^	8.28 × 10^−7^	0.44
RVSV (mL)	−0.021^a^	−0.025^a^	−0.023^a^	−0.009
	[−0.030, −0.011]	[−0.040, −0.010]	[−0.038, −0.008]	[−0.028, 0.009]
	1.46 × 10^−5^	1.43 × 10^−3^	2.32 × 10^−3^	0.32

Effect sizes represent standardised change in CMR parameter per 1-SD increase in neuroticism score with 95% confidence intervals and *P*-values. Models adjusted for age, sex, BMI, Townsend deprivation score, smoking, diabetes, hypertension, hypercholesterolaemia, alcohol use, exercise, and education. AoD, aortic distensibility; ASI, arterial stiffness index; CMR, cardiovascular magnetic resonance; LVEDV, left ventricular end-diastolic volume; LVGFI, left ventricular global function index; LVGLS, left ventricular global longitudinal strain; LVM, left ventricular mass; LVSV, left ventricular stroke volume; RVEDV, right ventricular end-diastolic volume; RVSV, right ventricular stroke volume; SD, standard deviation.

^a^Indicates statistical significance.

**Figure 1:**
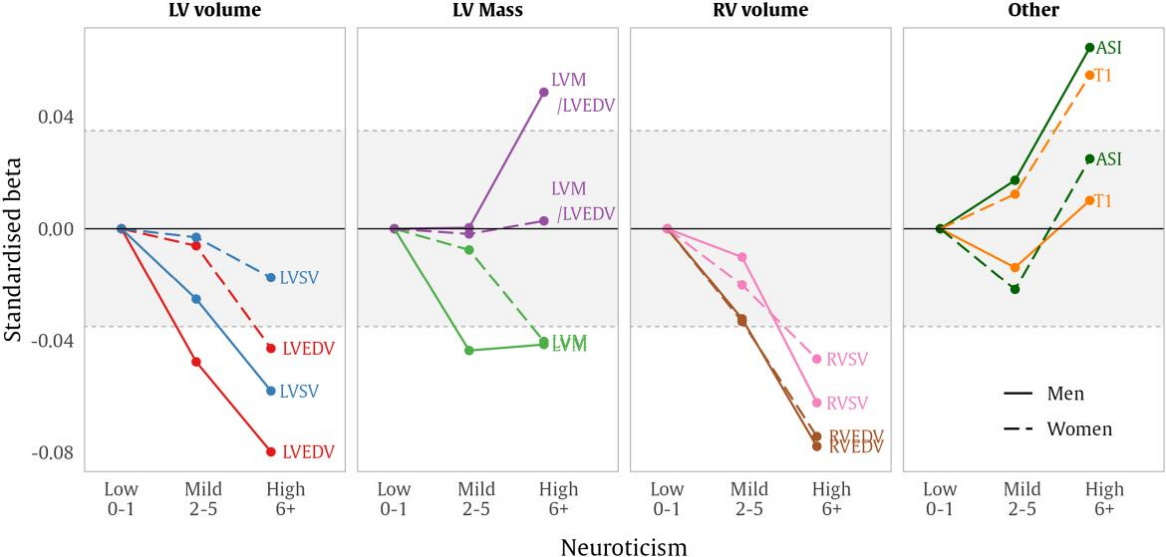
CMR parameter differences by neuroticism tertile. Points are standardised betas showing SD difference in CMR associated with neuroticism category, compared to the Low neuroticism group. Results for men are shown with a solid line, results for women are shown with a dashed line. The area shaded with grey indicates effects that are not significantly different from the Low neuroticism group. Models adjusted for age, sex, BMI, Townsend deprivation score, smoking, diabetes, hypertension, hypercholesterolaemia, alcohol use, exercise, and education. ASI, arterial stiffness index; CMR, cardiovascular magnetic resonance; LVEDV, left ventricular end-diastolic volume; LVM, left ventricular mass; LVSV, left ventricular stroke volume; RVEDV, right ventricular end-diastolic volume; RVSV, right ventricular stroke volume; SD, standard deviation.

There were no significant associations between neuroticism scores and LV global longitudinal strain, LV global function index, or AoD. These results are also reported as standardised changes in CMR parameters by neuroticism tertile (*Figure 1*, Supplementary data online, *Table S2*) and absolute changes in CMR parameters by neuroticism tertile in Supplementary data online, *Table S3*.

### Associations between neuroticism and CMR metrics for men

For men, in fully adjusted models, each 1SD increase in neuroticism score was associated with smaller LVEDV [−0.033 (−0.048 to −0.018)] and RVEDV [−0.034 (−0.049 to −0.019)], lower LVSV [−0.024 (−0.039 to −0.009)] and RVSV [−0.025 (−0.040 to −0.010)], smaller LVM [−0.019 (−0.033 to −0.005)], and greater concentricity [LVM/LVEDV, 0.018 (0.003 to 0.033)]. Furthermore, higher neuroticism score was associated with greater ASI [0.030 (0.012 to 0.047)] (*Table 4*).

There were no significant associations between neuroticism scores and native T1 values, LV global longitudinal strain, LV global function index, or AoD. These results are also reported as standardised changes in CMR parameters by neuroticism tertile (see Supplementary data online, *Table S2*) and absolute changes in CMR parameters by neuroticism tertile in Supplementary data online, *Table S3*.

### Associations between neuroticism and CMR metrics for women

For women, in fully adjusted models, each 1SD increase in neuroticism score was associated with smaller LVEDV [−0.024 (−0.039 to −0.009)] and RVEDV [−0.037 (−0.051 to −0.022)], lower RVSV [−0.023 (−0.038 to −0.008)], and smaller LVM [−0.018 (−0.032 to −0.004)]. Furthermore, higher neuroticism score was associated with greater native T1 values [0.030 (0.014 to 0.046)] (*Table 4*).

There were no significant associations between neuroticism scores and LVSV, LVM/LVEDV, ASI, LV global longitudinal strain, LV global function index, or AoD. These results are also reported as standardised changes in CMR parameters by neuroticism tertile (see Supplementary data online, *Table S2*) and absolute changes in CMR parameters by neuroticism tertile in Supplementary data online, *Table S3*.

## Discussion

In this large population-based cohort study of over 30 000 adults with detailed standardised CMR, we identified significant associations between an individual’s neuroticism score and altered cardiac morphology and function, independent of multiple demographic and clinical vascular risk factors. Specifically, greater neuroticism scores were linked to smaller, poorer functioning ventricles with lower LV mass, a greater concentric LV remodelling, greater myocardial fibrosis (higher myocardial native T1), and higher arterial stiffness. These relationships are consistent with phenotypic alterations seen in cardiac aging. These associations appeared to be more prominent in men compared to women. Thus, greater neuroticism is linked to cardiovascular phenotypic alterations indicative of greater cardiac aging.

Negative emotional states including neuroticism have been linked with higher cardiac risk and mortality.^8–11^ To the best of our knowledge, our study is the first to assess the relationship between neuroticism and cardiovascular phenotypes. With respect to cardiac morphology, we found significant associations between higher neuroticism scores and reduced ventricular volumes, function, LV mass, and increased myocardial fibrosis. Previous studies have shown an association between depressive symptoms and diastolic dysfunction. Using echocardiography, Kim *et al*.^34^ showed moderate to severe depression was linked to lower early diastolic velocity determined by tissue Doppler imaging. Gustad *et al*.^35^ identified a relationship between previous and repeated depression symptoms and e’, an echocardiographic index of diastolic function. These findings were corroborated by Santiago *et al*.^36^ who found an inverse association between clinically significant depressive symptoms and septal e’ velocity. Our results extend existing knowledge by identifying novel changes in cardiac morphology and function in individuals with neuroticism personality traits using reference standard CMR imaging across the largest dataset examined. Importantly, we demonstrate that relationships between unhealthy cardiac remodelling exist not only in individuals with clinically diagnosed mental illness or clinically significant symptoms, but also in the wider population of individuals who are mostly free from clinical mental health diseases.

Our analysis also identified an association between greater neuroticism scores and increased arterial stiffness. Similar results were demonstrated in a substudy of the Baltimore Longitudinal Study of Aging where a relationship was observed between increased anger suppression, assessed using the Spielberger Anger Expression Inventory, and carotid arterial stiffness, quantified using a function of carotid distensibility.^13^ Additionally, using pulse wave velocity, Midei *et al*.^12^ demonstrated an association between attachment anxiety and total hostility and increased arterial stiffness. Our findings add to the evidence that negative emotional states are linked to increased arterial stiffness.

Furthermore, higher neuroticism scores were associated with worse LV function alongside increased concentricity and arterial stiffness in men compared to women, whereas higher neuroticism scores were linked with greater myocardial fibrosis in women. Previous work has demonstrated men are more likely to be diagnosed with CAD in comparison to women,^17^ while women have a higher propensity to develop heart failure.^19,20^ Our findings add to the literature that sex plays a role in cardiovascular phenotypes and highlights the need for more research to elucidate sex differences pertaining to neurotic personality traits.

The mechanisms driving these morphological changes remain to be delineated. In our study, higher neuroticism scores were associated with a pattern of CMR-derived metrics comparable to that seen with cardiovascular aging, with evidence of possible early, pre-clinical, adverse cardiac remodelling. This implies greater neuroticism scores are linked with a premature aging effect on the ventricles. These findings provide possible insight into the processes leading to adverse cardiovascular outcomes in those with higher neuroticism scores, and emphasises the potential importance of assessing neuroticism as a marker of cardiovascular aging and risk.

This study supports the idea of personalising individual healthcare by detecting novel factors that could be considered as part of comprehensive cardiovascular risk assessment,^37,38^ such as neuroticism, and accordingly implementing protective lifestyle and health-based interventions with the aim of reducing their CVD risk profile and thereby prevent future adverse cardiovascular events.^39–41^

## Limitations

The assessment of neuroticism at a single time point may not fully represent an individual’s mental status. As we consider neuroticism traits in people who are mostly free from mental health diagnoses, we may not capture more pronounced cardiovascular consequences that are more likely in individuals with clinical psychiatric illnesses. Studies examining relationships with self-reported personality traits are liable to reporting biases, in particular social desirability bias. The use of total neuroticism questionnaire score does not consider potential differential importance of individual components of the scale. Limited ethnic diversity in the UK Biobank precludes examination of differential relationships across ethnic groups. The observational nature of the study means that we cannot exclude residual or unmeasured confounding or infer causality.

## Conclusions

Greater tendency towards neuroticism personality traits is linked to unhealthy cardiovascular remodelling patterns, independent of traditional vascular risk factors. The general pattern of associations is consistent in men and women, with more robust relationships observed in men. Our findings highlight the link between mental health and cardiovascular health and lend support to individual and population level strategies to promote mental wellbeing.

## Supplementary Material

jead166_Supplementary_Data

## Data Availability

This research was conducted using the UK Biobank resource under access application 2964. UK Biobank will make the data available to all bona fide researchers for all types of health-related research that is in the public interest, without preferential or exclusive access for any persons. All researchers will be subject to the same application process and approval criteria as specified by UK Biobank. For more details on the access procedure, see the UK Biobank website: http://www.ukbiobank.ac.uk/register-apply.
