# Supplementary material for: Neuroticism personality traits are linked to adverse cardiovascular phenotypes in the UK Biobank
Source: Eur Heart J Cardiovasc Imaging. 2023 Jul 13;24(11):1460–7. doi: 10.1093/ehjci/jead166 (PMC10610755; doi:10.1093/ehjci/jead166)
Supplement: jead166_Supplementary_Data [file jead166_supplementary_data.docx]

**Supplementary Figure 1. Distribution of neuroticism scores**

IQR, interquartile range; SD, standard deviation.

|  | **Questions** |
| --- | --- |
| 1 | Does your mood often go up and down? |
| 2 | Do you ever feel 'just miserable' for no reason? |
| 3 | Are you an irritable person? |
| 4 | Are your feelings easily hurt? |
| 5 | Do you often feel 'fed-up'? |
| 6 | Would you call yourself a nervous person? |
| 7 | Are you a worrier? |
| 8 | Would you call yourself tense or 'highly strung'? |
| 9 | Do you worry too long after an embarrassing experience? |
| 10 | Do you suffer from 'nerves'? |
| 11 | Do you often feel lonely? |
| 12 | Are you often troubled by feelings of guilt? |

**Supplementary Table 1. EPQ-R-S neuroticism scale**

EPQ-R-S, Eysenck Personality Questionnaire-Revised Short Form.

**Supplementary Table 2. CMR parameter differences by neuroticism tertile**

| **Metric** | **Neuroticism** | **Whole cohort** | **Men** | **Women** |
| --- | --- | --- | --- | --- |
| LVEDV (mL) | 2 to 5 | -0.03* [-0.05, -0.01]  0.01 | -0.05* [-0.08, -0.01]  8.57x10^-3^ | -0.01 [-0.04, 0.03]  0.73 |
|  | 6+ | -0.05* [-0.07, -0.03]  8.10x10^-7^ | -0.08* [-0.12, -0.04]  3.07x10^-5^ | -0.04* [-0.08, -0.01]  0.02 |
| LVSV (mL) | 2 to 5 | -0.02 [-0.04, 0.01]  0.17 | -0.03 [-0.06, 0.01]  0.17 | -0.00 [-0.04, 0.03]  0.87 |
|  | 6+ | -0.04* [-0.06, -0.01]  2.69x10^-3^ | -0.06* [-0.10, -0.02]  2.69x10^-3^ | -0.02 [-0.05, 0.02]  0.34 |
| LVM (g) | 2 to 5 | -0.02* [-0.04, -0.00]  0.03 | -0.04* [-0.08, -0.01]  9.95x10^-3^ | -0.01 [-0.04, 0.03]  0.66 |
|  | 6+ | -0.03* [-0.05, -0.01]  3.57x10^-4^ | -0.04* [-0.08, -0.01]  0.02 | -0.04* [-0.07, -0.01]  0.02 |
| LVM/LVEDV (g/mL) | 2 to 5 | 0.00 [-0.02, 0.03]  0.75 | 0.00 [-0.04, 0.04]  0.98 | -0.00 [-0.04, 0.03]  0.92 |
|  | 6+ | 0.02 [-0.00, 0.05]  0.06 | 0.05* [0.01, 0.09]  9.62x10^-3^ | 0.00 [-0.03, 0.04]  0.87 |
| LVGLS (%) | 2 to 5 | 0.00 [-0.03, 0.04]  0.89 | -0.03 [-0.08, 0.02]  0.31 | 0.03 [-0.02, 0.08]  0.24 |
|  | 6+ | 0.03 [-0.00, 0.07]  0.08 | 0.03 [-0.03, 0.08]  0.31 | 0.04 [-0.01, 0.09]  0.09 |
| LVGFI (%) | 2 to 5 | -0.01 [-0.04, 0.02]  0.64 | 0.02 [-0.03, 0.06]  0.42 | -0.03 [-0.07, 0.02]  0.20 |
|  | 6+ | 0.01 [-0.02, 0.04]  0.40 | -0.01 [-0.06, 0.04]  0.63 | 0.03 [-0.02, 0.07]  0.20 |
| Native T1 (ms) | 2 to 5 | -0.00 [-0.03, 0.02]  0.90 | -0.01 [-0.05, 0.03]  0.48 | 0.01 [-0.03, 0.05]  0.52 |
|  | 6+ | 0.03* [0.01, 0.06]  0.02 | 0.01 [-0.03, 0.05]  0.63 | 0.06* [0.02, 0.09]  4.88x10^-3^ |
| AoD (10-3/mmHg) | 2 to 5 | -0.00 [-0.05, 0.02]  0.52 | -0.00 [-0.05, 0.05]  0.99 | -0.03 [-0.08, 0.02]  0.20 |
|  | 6+ | -0.00 [-0.03, 0.04]  0.52 | -0.00 [-0.05, 0.05]  0.91 | 0.00 [-0.05, 0.05]  0.98 |
| ASI (m/s) | 2 to 5 | -0.00 [-0.03, 0.03]  0.91 | 0.02 [-0.02, 0.06]  0.40 | -0.02 [-0.06, 0.02]  0.30 |
|  | 6+ | 0.04* [0.02, 0.07]  3.28x10^-3^ | 0.07* [0.02, 0.11]  3.10x10^-3^ | 0.03 [-0.02, 0.07]  0.24 |
| RVEDV (mL) | 2 to 5 | -0.03* [-0.05, -0.01]  7.36x10^-3^ | -0.03 [-0.07, 0.00]  0.07 | -0.03 [-0.07, 0.00]  0.06 |
|  | 6+ | -0.06* [-0.08, -0.04]  8.77x10^-9^ | -0.08* [-0.12, -0.04]  4.29x10^-5^ | -0.07* [-0.11, -0.04]  3.63x10^-5^ |
| RVSV (mL) | 2 to 5 | -0.01 [-0.04, 0.01]  0.20 | -0.01 [-0.05, 0.03]  0.58 | -0.02 [-0.06, 0.02]  0.27 |
|  | 6+ | -0.05* [-0.07, -0.02]  5.88x10^-5^ | -0.06* [-0.10, -0.02]  1.31x10^-3^ | -0.05* [-0.08, -0.01]  0.01 |

Standardised betas with 95% confidence intervals and p-values showing SD difference in CMR associated with neuroticism category, compared to the Low neuroticism group. Estimates are from linear regression models adjusted for age, sex, BMI, Townsend deprivation score, smoking, diabetes, hypertension, hypercholesterolaemia, alcohol use, exercise, and education. AoD, aortic distensibility; ASI, arterial stiffness index; CMR, cardiovascular magnetic resonance; LVEDV, left ventricular end-diastolic volume; LVGFI, left ventricular global function index; LVGLS, left ventricular global longitudinal strain; LVM, left ventricular mass; LVSV, left ventricular stroke volume; RVEDV, right ventricular end-diastolic volume; RVSV, right ventricular stroke volume; SD, standard deviation. * indicates statistical significance.

**Supplementary Table 3. Absolute CMR parameter differences by neuroticism tertile**

| **Metric** | **Neuroticism** | **Whole cohort** | **Men** | **Women** |
| --- | --- | --- | --- | --- |
| LVEDV (mL) | 2 to 5 | -0.88* [-1.57, -0.20]  0.01 | -1.50* [-2.62, -0.38]  0.01 | -0.14 [-0.93, 0.65]  0.73 |
|  | 6+ | -1.77* [-2.47, -1.07]  8.10x10^-7^ | -2.52* [-3.70, -1.33]  3.07x10^-5^ | -0.97* [-1.76, -0.18]  0.02 |
| LVSV (mL) | 2 to 5 | -0.30 [-0.73, 0.13]  0.17 | -0.49 [-1.18, 0.21]  0.17 | -0.04 [-0.56, 0.47]  0.87 |
|  | 6+ | -0.68* [-1.12, -0.23]  2.70x10^-3^ | -1.12* [-1.85, -0.39]  2.70x10^-3^ | -0.25 [-0.77, 0.26]  0.34 |
| LVM (g) | 2 to 5 | -0.41 [-0.78, -0.04]  0.03 | -0.81* [-1.42, -0.19]  0.01 | -0.09 [-0.51, 0.32]  0.66 |
|  | 6+ | -0.70* [-1.08, -0.31]  3.57x10^-4^ | -0.77 [-1.41, -0.12]  0.02 | -0.50 [-0.91, -0.09]  0.02 |
| LVM/LVEDV (g/mL) | 2 to 5 | 0.03 [-0.17, 0.24]  0.75 | 0.00 [-0.32, 0.32]  0.98 | -0.01 [-0.28, 0.25]  0.92 |
|  | 6+ | 0.21 [-0.01, 0.42]  0.06 | 0.45* [0.11, 0.79]  9.62x10^-3^ | 0.02 [-0.24, 0.28]  0.87 |
| LVGLS (%) | 2 to 5 | 0.01 [-0.09, 0.10]  0.89 | -0.07 [-0.20, 0.06]  0.31 | 0.08 [-0.05, 0.21]  0.24 |
|  | 6+ | 0.08 [-0.01, 0.18]  0.08 | 0.07 [-0.07, 0.21]  0.31 | 0.11 [-0.02, 0.25]  0.09 |
| LVGFI (%) | 2 to 5 | -0.05 [-0.24, 0.15]  0.64 | 0.11 [-0.16, 0.38]  0.42 | -0.18 [-0.46, 0.10]  0.20 |
|  | 6+ | 0.09 [-0.11, 0.28]  0.40 | -0.07 [-0.36, 0.22]  0.63 | 0.18 [-0.10, 0.46]  0.20 |
| Native T1 (ms) | 2 to 5 | -0.06 [-0.96, 0.84]  0.90 | -0.45 [-1.70, 0.80]  0.48 | 0.42 [-0.86, 1.70]  0.52 |
|  | 6+ | 1.09* [0.17, 2.01]  0.02 | 0.33 [-0.99, 1.64]  0.63 | 1.85* [0.56, 3.13]  4.88x10^-3^ |
| AoD (10-3/mmHg) | 2 to 5 | -0.03 [-0.09, 0.02]  0.27 | -0.00 [-0.07, 0.07]  0.99 | -0.06 [-0.14, 0.03]  0.20 |
|  | 6+ | 0.00 [-0.06, 0.06]  0.99 | -0.00 [-0.08, 0.07]  0.91 | 0.00 [-0.08, 0.09]  0.98 |
| ASI (m/s) | 2 to 5 | -0.01 [-0.09, 0.08]  0.91 | 0.05 [-0.07, 0.17]  0.40 | -0.06 [-0.19, 0.06]  0.30 |
|  | 6+ | 0.13* [0.04, 0.22]  3.30x10^-3^ | 0.19* [0.06, 0.31]  3.10x10^-3^ | 0.07 [-0.05, 0.20]  0.24 |
| RVEDV (mL) | 2 to 5 | -0.99* [-1.72, -0.27]  7.36x10^-3^ | -1.08 [-2.26, 0.10]  0.07 | -0.80 [-1.64, 0.05]  0.64 |
|  | 6+ | -2.19* [-2.93, -1.44]  8.77x10^-9^ | -2.60* [-3.84, -1.35]  4.29x10^-5^ | -1.79* [-2.63, -0.94]  3.63x10^-5^ |
| RVSV (mL) | 2 to 5 | -0.29 [-0.74, 0.16]  0.20 | -0.20 [-0.92, 0.51]  0.58 | -0.30 [-0.83, 0.23]  0.27 |
|  | 6+ | -0.94* [-1.40, -0.48]  5.88x10^-5^ | -1.24* [-2.00, -0.48]  1.31x10^-3^ | -0.69* [-1.23, -0.16]  0.01 |

Non-standardised betas with 95% confidence intervals and p-values showing absolute difference in CMR associated with neuroticism category, compared to the Low neuroticism group. Estimates are from linear regression models adjusted for age, sex, BMI, Townsend deprivation score, smoking, diabetes, hypertension, hypercholesterolaemia, alcohol use, exercise, and education. AoD, aortic distensibility; ASI, arterial stiffness index; CMR, cardiovascular magnetic resonance; LVEDV, left ventricular end-diastolic volume; LVGFI, left ventricular global function index; LVGLS, left ventricular global longitudinal strain; LVM, left ventricular mass; LVSV, left ventricular stroke volume; RVEDV, right ventricular end-diastolic volume; RVSV, right ventricular stroke volume. * indicates statistical significance.
